# Transcription Factor Sp1 Promotes the Expression of Porcine *ROCK1* Gene

**DOI:** 10.3390/ijms17010112

**Published:** 2016-01-15

**Authors:** Ruirui Zhang, Xiaoting Feng, Mengsi Zhan, Cong Huang, Kun Chen, Xiaoyin Tang, Tingting Kang, Yuanzhu Xiong, Minggang Lei

**Affiliations:** 1 Key Laboratory of Swine Genetics and Breeding of Agricultural Ministry & Key Laboratory of Agricultural Animal Genetics, Breeding and Reproduction of Ministry of Education, College of Animal Science and Technology, Huazhong Agricultural University, Wuhan 430070, China; zhangruirui807870@163.com (R.Z.); xiaotingfeng1984@163.com (X.F.); zmsyx.2581@163.com (M.Z.); huangcong19880426@126.com (C.H.); kunchen1989@163.com (K.C.); wlifetxy@sina.com (X.T.); 13163228175@163.com (T.K.); 2 College of Life Science and Technology, Wuhan Bioengineering Institute, Wuhan 430070, China

**Keywords:** ROCK1, transcription factor, Sp1, regulation

## Abstract

Rho-associated, coiled-coil containing protein kinase 1 (*ROCK1*) gene plays a crucial role in maintaining genomic stability, tumorigenesis and myogenesis. However, little is known about the regulatory elements governing the transcription of porcine *ROCK1* gene. In the current study, the transcription start site (TSS) was identified by 5’-RACE, and was found to differ from the predicted one. The region in *ROCK1* promoter which is critical for promoter activity was investigated via progressive deletions. Site-directed mutagenesis indicated that the region from −604 to −554 bp contains responsive elements for Sp1. Subsequent experiments showed that *ROCK1* promoter activity is enhanced by Sp1 in a dose-dependent manner, whereas treatment with specific siRNA repressed *ROCK1* promoter activity. Electrophoretic mobility shift assay (EMSA), DNA pull down and chromatin immunoprecipitation (ChIP) assays revealed Sp1 can bind to this region. qRT-PCR and Western blotting research followed by overexpression or inhibition of Sp1 indicate that Sp1 can affect endogenous *ROCK1* expression at both mRNA and protein levels. Overexpression of Sp1 can promote the expression of *myogenic differentiation 1(MyoD), myogenin (MyoG), myosin heavy chain (MyHC)*. Taken together, we conclude that Sp1 positively regulates *ROCK1* transcription by directly binding to the *ROCK1* promoter region (from −604 to −532 bp) and may affect the process of myogenesis.

## 1. Introduction

As a downstream effector of the small GTP-binding protein Rho, rho-associated, coiled-coil containing protein kinase (*ROCK*) acts as a molecular switch controlling a variety of cellar functions, such as the regulation of stress fiber formation, actin polymerization and so on [1,2]. *ROCK1* and *ROCK2*, two isoforms of *ROCK*, have distinct roles and cannot be replaced by each other [3].

*ROCK1* participates in multiple biological and physiological processes [4,5]. Besides the important role in the progress of tumorigenesis, obesity, and inflammation [5,6,7], *ROCK1* also participates in the regulation of skeletal muscle [8,9]. Additionally, numerous elements such as RhoA, medicine, sex hormone, and nitric oxide can regulate the activity of ROCK1 [10,11,12].The activation of ROCK1 is necessary and sufficient to control glucose transport in myoblasts [13]. During myogenesis, *ROCK1* is reported to act as a negative regulator of the process [9]. *ROCK1* is required for myoblast proliferation, but prevents commitment to differentiation [8]. Despite the researchers increasing focus on the biological role of ROCK1 gene, little is known about the transcriptional regulation of porcine *ROCK1* gene. Therefore, it is crucial to elucidate the molecular mechanisms involved in its expression and transcriptional regulation.

Sp1, a member of the SP/KLF transcription factor family, is an important regulator in many tissues that binds to GC-rich motifs, which plays a key role in cellular functions [14,15]. It always works through binding to the promoter region of its target genes [16,17], and can activate or repress the transcription in response to physiological and pathological stimuli [18]. The promoter activity of rat *ROCK1* gene is reduced by Sp1, whereas it is enhanced by Sp6 in dental epithelial cells [19].

To investigate the transcriptional regulation of the *ROCK1* gene, we isolated the promoter of the porcine *ROCK1* gene, analyzed its upstream regulatory elements and revealed that transcription factor Sp1 directly binds to the core promoter region of *ROCK1* and stimulates its expression.

## 2. Results

### 2.1. Identification of the Promoter Region and Regulatory Elements of Porcine ROCK1 Gene.

**Figure 1:**
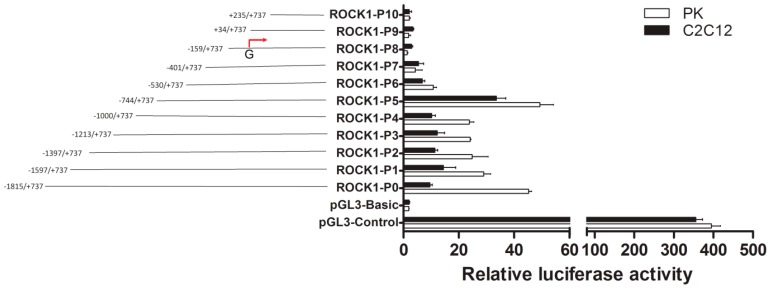
5’-Deletion analysis of the porcine *ROCK1* promoter activity. Schematic representation of the progressive deletions of porcine *ROCK1* 5’-flanking region in pGL3-Basic vector and the relative activities of *ROCK1* promoter corresponding to the progressive deletions. The predicted transcription start site (TSS, the red arrow in the figure) was set +1, differs from the TSS in NCBI database. The pGL3-control/basic vectors were used as positive/negative control, while pRL-TK was used as internal control. Data were expressed as means ± SD of three replicates.

A 2552-bp 5’-flanking region of porcine *ROCK1* gene was obtained and 11 progressive deletions were introduced upstream of the luciferase reporter gene. It was noticed that the *ROCK1*-P7 to P10 fragments had no activity according to negative control; an increase of activity was detected in P6 and P5, especially the P5 fragment (Figure 1), indicating that the region from P5 to P6 (−744 to −402 bp) was important for transcriptional activity (Figure 1).

In addition, differing from the predicted transcription start site (TSS), the TSS obtained by 5**’** RACE was located at −430 bp (Figure S1), suggesting the importance of the region from −744 to −402 bp. According to the TFsearch and the JASPAR database, three potential Sp1 binding sites (−604/−595 bp, −561/−554 bp and −543/−532 bp) were located in the region (Figure S1).

### 2.2. The Importance of Sp1 Binding Sites in Porcine ROCK1 Promoter

**Figure 2:**
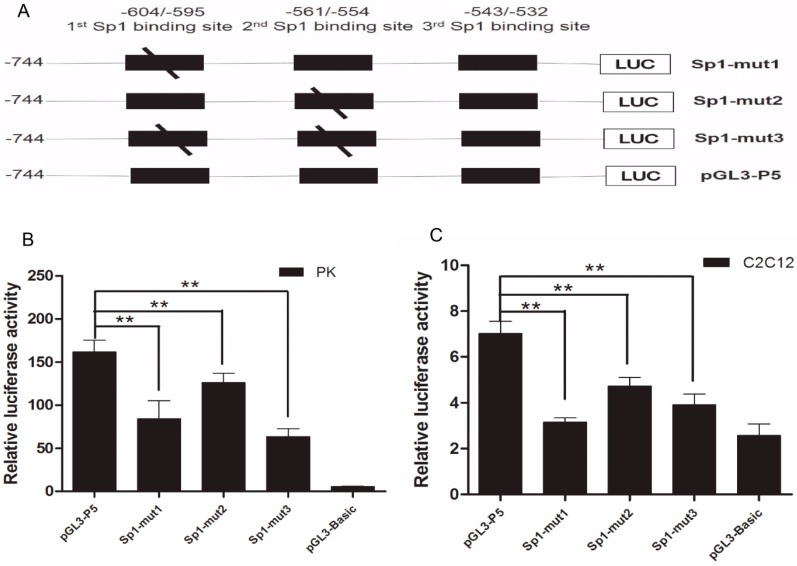
Site-directed mutation of Sp1 binding sites in *ROCK1*-P5 fragment. (**A**) Schematic structure of site-directed mutagenesis in the putative Sp1 binding sites (the black slash) of porcine *ROCK1* gene. LUC represents the *Luciferase* gene in the vectors; (**B**,**C**) Luciferase activity of site-directed mutagenesis in PK and C2C12 cells. Statistical differences of relative activities were analyzed in the same cells; ** *p* < 0.01, data were expressed as means ± SD of three replicates.

To functionally determine the importance of Sp1 binding sites, site-directed mutagenesis was performed (Figure 2A). The modification in −604/−595 bp and −561/−554 bp regions obviously blocked the Sp1-stimulated transcription activity (Figure 2B,C) in both PK and C2C12 cells. These results revealed that the two binding sites (located at −604/−595 bp and −561/−554 bp) are essential for *ROCK1* promoter activity.

### 2.3. Sp1 Binds to the Porcine ROCK1 Promoter in Vitro and in Vivo

**Figure 3:**
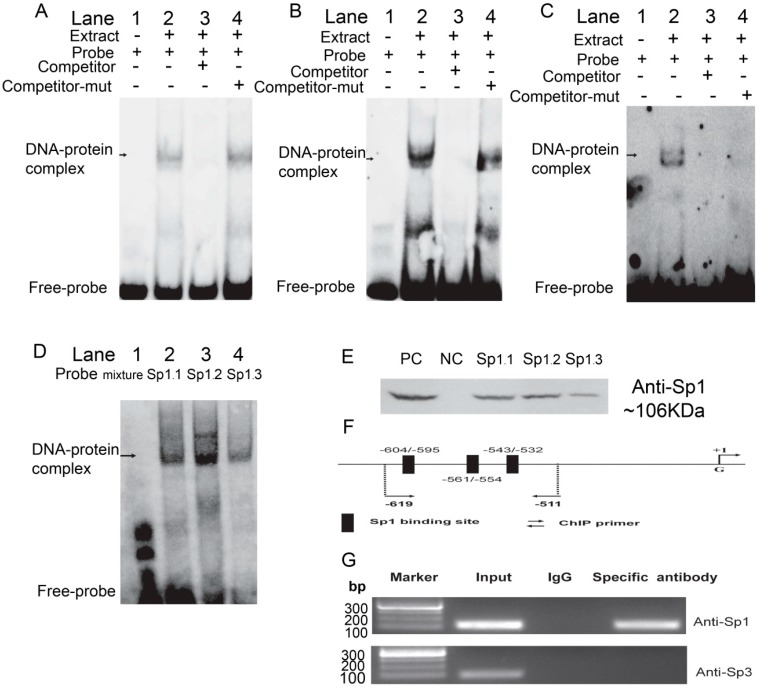
Binding of Sp1 with the *ROCK1*-P5 fragment was analyzed *in vitro* and *in vivo*. The first (**A**), the second (**B**) and the third (**C**) biotin-labeled probes were incubated with the NE of PK cells. Lane 1 was the negative control without NE; the reagents were incubated in the absence competitor probes in Lane 2 or in presence of 50× excess competitor (Lane 3)/competitor-mutant (Lane 4) probes, respectively; (**D**) The three probes were incubated with PK NE, respectively; (**E**) Proteins of PK extracted from DNA-pull down materials were detected by Western blot. The total non-denaturing proteins/Streptavidin MagneSphere^®^ Paramagnetic Particles were taken as positive/negative control (PC/NC). The three potential Sp1 binding sites were named as Sp1.1, Sp1.2, and Sp1.3 in (**D**,**E**). The competitor/competitor-mutant probes were 50-fold excess and arrows indicated the specific DNA-protein complex bands; (**F**) Schematic diagram of the Sp1 binding sites in the porcine *ROCK1*-P5 fragment; (**G**) ChIP assay of Sp1 binding to porcine *ROCK1*-P5 fragment in PK cells. The *in vivo* interaction of Sp1 and Sp3 with porcine *ROCK1* promoter was determined by ChIP assay, in which Normal mouse IgG was used as negative control. DNA isolated from immunoprecipitated materials was used for PCR amplification, whereas total chromatin was used as input (positive control). The antibodies used in ChIP assay were listed in the right of the figure and the corresponding amplification product obtained here was 107 bp.

The EMSA and DNA pull down assays were used to determine whether transfection factor Sp1 could bind to promoter region of porcine *ROCK1* gene *in vitro*. As shown Figure 3A, the incubation of Nuclear extract (NE) from PK cells with Sp1 probe 1 gave rise to the formation of a DNA–protein complex (Lane 2), which could be observed with competitor-mut probe (Lane 4), but not with competitor probe (Lane 3). Moreover, DNA-protein bands of the other two probes were also detected (Figure 3B,C), and when incubated with the same NE, each site showed a different binding ability, whereas the molecular size of the three DNA–protein complexes were similar (Figure 3D). Moreover, the proteins obtained from DNA pull down assay were detected using anti-Sp1 by Western blotting (Figure 3E), suggesting the protein bound to *ROCK1* promoter region was exactly the transcription factor Sp1. Similar results of EMSA and DNA pull down were observed in NE of pig longissimus dorsi muscle (LM) (Figure S2A–E).

To determine the *in vivo* binding of Sp1 and *ROCK1* promoter, ChIP analysis was performed in PK cells. The position information of the ChIP-PCR primers is shown in Figure 3F where the three sites are considered as a cluster. DNA fragment of the expected size was obtained when anti-Sp1 was added (Figure 3G). However, when antibody for Sp3 (another member the SP/KLF transcription factor family) was used, the expected DNA fragment did not appear (Figure 3G). The results showed that Sp1, rather than Sp3, directly interacted with the *ROCK1* promoter.

Taken together, these findings suggested that the proximal Sp1 binding sites of the *ROCK1* promoter region were capable of binding to Sp1 protein both *in vitro* and *in vivo*.

### 2.4. Sp1 Stimulates ROCK1 Gene Expression

**Figure 4:**
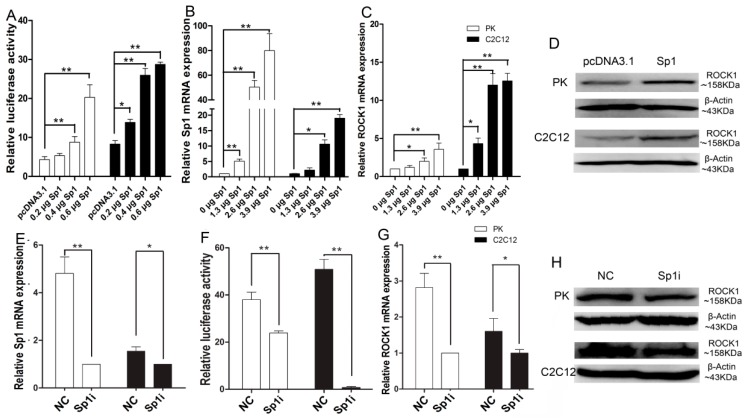
Sp1 stimulates the expression of porcine *ROCK1* gene. (**A**) Over-expression of *Sp1* up-regulated *ROCK1* luciferase activity; (**B**) Over-expression efficacy of Sp1; (**C**,**D**) Over-expression of Sp1 stimulated *ROCK1* expression at mRNA and protein level; (**E**) The interferences efficacy of siRNA; (**F**) Suppressing *Sp1* reduced the *ROCK1* promoter activity; (**G**,**H**) Inhibition of *Sp1* suppressed *ROCK1* expression at mRNA and protein level. The amount of plasmid was kept constant by the addition of pcDNA3.1 (+) vector. The data were obtained both in PK and C2C12 cells and expressed as means ± SD of three replicates. * *p* < 0.05, ** *p* < 0.01.

According to the prediction of *cis*-acting elements, the overexpression and inhibition of *Sp1* were performed. When overexpressing *Sp1* both in PK and C2C12 cells, the *ROCK1*-P5 activity was significantly increased depending on the amount of *Sp1* (Figure 4A,B). Furthermore, overexpression of *Sp1* significantly promoted *ROCK1* expression at mRNA level (*p* < 0.05), which was also dependent on the amount of Sp1 (Figure 4C). Meanwhile, a similar tendency was observed at protein level (Figure 4D). Additionally, when inhibiting *Sp1* by specific siRNAs, a clear decrease of *ROCK1* promoter activity and the expression of *ROCK1* were observed both in PK and C2C12 cells (Figure 4F–H). Taken together, our data showed that Sp1 acted as a positive regulator of *ROCK1* transcription.

### 2.5. Sp1 Stimulates the Process of Myogenesis

**Figure 5:**
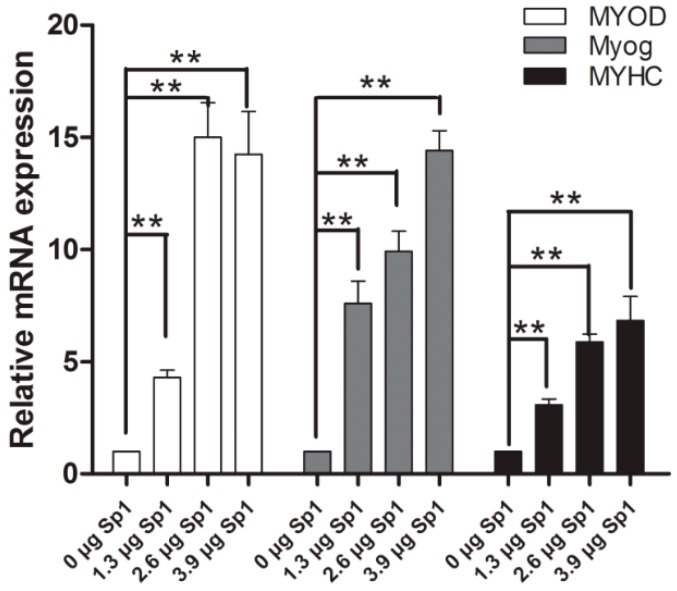
*Sp1* promotes the process of myogenesis. Over-expression of *Sp1* significantly stimulated *MyoD*, *MyoG*, *MyHC* mRNA expression in C2C12 cells, ** *p* < 0.01.

To determine whether *ROCK1* affects the process of myogenesis via *Sp1*, *Sp1* was forced expressed in C2C12 cells. Overexpression of *Sp1* results in the up-regulation of *MyoD, MyoG,* and *MyHC* at mRNA level (Figure 5), indicating that Sp1 can stimulate the process of myogenesis.

## 3. Discussion

The promoter region is reported to regulate the transcription initiation and the expression of gene which is critical for the regulation of gene expression [20]. Our experimental analysis indicated that the 5’-flanking region from −744 to −402 bp of porcine *ROCK1* gene significantly affected the promoter activity (Figure 1 and Figure 2), suggesting that the core promoter is located in this region and the regulatory elements of this region may enhance the promoter activity of *ROCK1*.

The *in silico* analysis of the promoter region of porcine *ROCK1* reveals that this region exhibits an extremely high GC content (up to 54.82%), particularly the core promoter region (from −744 to −402 bp, accounting for 74.72%), and the region contains several GC boxes. Further analysis indicated the core promoter region of porcine *ROCK1* contains several potential binding sites for Sp1, in line with the report that Sp1 binding sites often occur as multiple repeats [21]. Sp1, a regulator in many tissues, plays a vital role in numerous cellular functions such as apoptosis and invasion [22,23] and usually regulates the expression of its target gene via binding to their promoters [24]. It often works through binding to GC-rich decanucleotide recognition elements (GC boxes) with a consensus sequence 5’-(G/T)GGGCGG(G/A)(G/A)(G/T)-3’ [25,26]. The EMSA, DNA pull down and ChIP assays revealed that Sp1 does bind to the *ROCK1* promoter directly and these interactions are important determinants of basal promoter activity. Furthermore, the specific DNA–protein complexes detected by EMSA indicate that Sp1 can bind independently to each potential GC boxes, in accordance with former research [27]. Previous studies indicate that *Sp1* and *Sp3* can cooperate or compete to regulate the expression of target genes [28,29]. The presence of expected amplification products when DNA precipitated with Sp1 antibody and the absence of expected amplification products when DNA precipitated with Sp3 antibody indicate that Sp1, not Sp3, directly binds to ROCK promoter to transcriptionally regulate the expression of *ROCK1*.

*Sp1* can promote or suppress the expression of its target gene [18]. The significant changes of *ROCK1* promoter activity and the endogenous *ROCK1* expression when overexpressing or inhibiting *Sp1* indicate that *Sp1* can stimulate the expression of *ROCK1* via the regulation of transcription activity, different from the previous report that Sp1 reduces the promoter activity of rat *ROCK1* gene in dental epithelial cells [19].

*ROCK1* gene has been implicated in the regulation of skeletal muscle growth [30,31]. Several studies via miRNA, overexpression or inhibition of *ROCK1* have demonstrated that *ROCK1* acts as a negative regulator in the myogenic process [8,9,32]. Mfy5 (myogenic factor 5), Mfy6 (MRF4), MyoD (myogenic differentiation) and myogenin (MyoG) are members of the myogenic regulatory factors (MRFs), and play crucial roles in the complex process of skeletal myogenesis, including commitment and proliferation, muscle fiber formation, and postnatal maturation and muscle function [33,34]. Myosin heavy chain (MyHC) is the essential component of myosin, the most abundant contractile molecule in mammalian skeletal muscles [35]. When forced expression of *Sp1* occurs, the significant increase of *MyoD*, *MyoG*, and *MyHC* in C2C12 cells suggests the significant role of Sp1 in regulating myoblasts differentiation, implying that *ROCK1* might participate in or partly inhibit the regulation of myoblasts’ differentiation via Sp1. Additionally, *Sp1* can affect the phosphorylation of multiple genes, which can further affect their functions [36,37]. The function of *ROCK* will be changed when its activity was modified [38]. Therefore, further research needs to be performed to determine whether Sp1 can affect the phosphorylation of porcine *ROCK1* gene.

Taken together, Sp1 acts as a critical regulatory factor for porcine *ROCK1* transcription and may regulate the development of pig skeletal muscle via Sp1-*ROCK1*-MRFs pathway, thus providing a novel regulation mechanism of porcine *ROCK1* and myogenesis.

## 4. Materials and Methods

### 4.1. Animals

Pigs (*S. scrofa*) used for this study were obtained from Jingpin pig station of Huazhong Agricultural University (Wuhan, China). All of the studies involving animals were conducted according to the regulation (No. 5 proclamation of the Standing Committee of Hubei People’s Congress) approved by the Standing Committee of Hubei People’s Congress, China. The sample collection was approved by the Ethics Committee of Huazhong Agricultural University with the permit number No. 30700571 for this study. The animals humanely sacrificed as necessary to ameliorate suffering. The methods were carried out in accordance with the approved guidelines. Four blood samples were preparation for genomic DNA and protein samples of the longissimus dorsi muscle (LM) were collected from every 60-day old Yorkshire pigs (3 pigs in total).

### 4.2. In Silico Sequence Analysis

The 2552 bp 5’up-stream sequence of porcine *ROCK1* gene was obtained from NCBI (http://www.ncbi.nlm.nih.gov). The promoter region was predicted by Proscan (http://www-bimas.cit.nih.gov/) and Sp1 binding sites were predicted by IFSEARCH (http://www.cbrc.jp/research/db/TFSEARCH.html) and the JASPAR database (http://jaspar.genereg.net/).

### 4.3. Rapid Amplification of 5’cDNA Ends (5’-RACE)

The LM of 60-day Yorkshire was used for RNA isolation, with total RNA from mouse heart provided in the kit as positive control. 5’-RACE was performed using the SMARTer™ RACE cDNA synthesis kit (Clontech, Shiga, Japan) according to the manufacturer's instructions and the primers were listed in Table S1.

### 4.4. Quantitative Real-Time Polymerase Chain Reaction (qRT-PCR)

Total-RNA was extracted with Total RNA isolation Kit (Omega, Bienne, Switzerland) and the cDNA was synthesised as previously described [39]. Subsequently, the expression was measured by qRT-PCR in LightCycler480 (Roche, Basel, Switzerland), using the gene-specific primers (Table S1). The *HPRT, eEF*γ, and *PPIA* were selected as housekeeping genes for PK cells [39], while β*-Actin* was used for C2C12 cells.

### 4.5. Plasmids’ Construction, Cell Culture, Transfection and Analysis

Serial deletions of porcine *ROCK1* 5’-flanking genomic region were amplified and denoted as *ROCK1*-P0-P10-Luc. Subsequently, the recombinant fragments were digested and inserted into pGL3-Basic vector (Promega, Madison, WI, USA). The overexpression plasmids were created by inserting the coding sequence (CDS) of porcine *Sp1* gene into the pcDNA3.1 (+) vector (Invitrogen, Cashman, CA, USA). All constructs were sequenced for verification. Primers used for amplification are listed in Table S2.

The PK (pig kidney cell line) and C2C12 (mouse myoblast cell line) cells were maintained at 37 °C in humidified 5% CO_2_ atmosphere in Dulbecco’s modified Eagle medium (Hyclone, Logan, UT, USA) supplemented with 10% fetal bovine serum (Gibico, New York, NY, USA). Cells were seeded in proper plates and cultured overnight. Then, the cells were transfected using Lipofectamine 2000 (Invitrogen). Site-directed mutagenesis of the *ROCK1***-**P5 (−744/+737)-Luc construct was performed by using the MutanBEST Kit (Takara, Tokyo, Japan) and specific primers (Table S3). Twenty-four hours after transfection, the luciferase activity was measured with PerkinElmer 2030 Multilabel Reader (PerkinElmer, Boston, MA, USA).

### 4.6. Electrophoretic Mobility Shift Assay (EMSA)

Nuclear extract (NE) of PK cells and LM of pig were extracted with Nucleoprotein Extraction Kit (Beyotime, Shanghai, China). Sequence specific probes (Sangon, Shanghai, China) were synthesized and annealed into double strands. The DNA binding ability was detected by EMSA with Scientific Light-Shift EMSA KIT (Thermo, Grand Island, NY, USA).

Briefly, proper component was added to the reaction, in which 20 fmol of Biotin-labeled oligonucleotides were added, the control group was supplemented with 50-fold excess of competitor/competitor-mut oligonucleotides. After incubation, the mixtures were conducted on polyacrylamide gels and transferred onto nylon membrane and analyzed with GE ImageQuant LAS4000 mini (GE-Healthcare, Little Chalfont, UK). Details of the oligonucleotide probes are shown in Table S3.

### 4.7. Chromatin Immunoprecipitation (ChIP) Assay

To measure the binding activity of Sp1 *in vivo*, ChIP assay was conducted with the EZ-ChIP™ Kit (Millipore, Bedford, MA, USA) according to the manufacturer’s protocol in PK cells. Briefly, DNA-protein complex were cross-linked and neutralized. After sonication, fragmented chromatin was added into ChIP dilution buffer, and incubated overnight with anti-Sp1 (Abcam, ab13370, Rabbit polyclonal antibody)/anti-Sp3 (Santa Cruz, sc-644x, Rabbit polyclonal antibody). A Normal Mouse IgG was added as negative control antibody. Immunoprecipitated products were collected after incubation with Protein A + G coated magnetic beads. The bound chromatin was eluted and digested with proteinase K, then the DNA was purified for PCR analysis (the primers are listed in Table S1).

### 4.8. DNA Pull down Assay

Non-denaturing proteins of PK cells and LM of the pig were extracted by Non-denaturing Lysis Buffer (Sangon). Later, we typically bonded non-denaturing proteins and biotin-labeled DNA probes by rotation, which were then supplemented with Streptavidin MagneSphere^®^ Paramagnetic Particles (Promega). Then, the reactions were further rotated and washed. Later, DNA-bound proteins were collected with 10% SDS (sodium dodecyl sulfate, sodium salt) and analyzed by Western blotting, taking non-denaturing proteins/Streptavidin MagneSphere^®^ Paramagnetic Particles as positive/negative control.

### 4.9. RNA Interference

Small interference fragments (siRNA) for *Sp1* were synthesized (GenePharma, Nanjing, China) and transfected according to the manufacturer’s instructions. Sus-Sp1-siRNA: GCGGCAAAGUAUAUGGCAATT and mus-Sp1-siRNA: UGAGAACAGCAACAACUCCTT were used in PK or C2C12 cells, respectively.

### 4.10. Western Blotting

Samples were heated in SDS buffer, separated by SDS-PAGE and transferred to PVDF (polyvinylidene fluoride) membranes. Then, the membranes were blocked and separately probed with anti-ROCK1 (Abcam, Cambridge, MA, USA, ab134181), and anti-Sp1 (Abcam, ab13370) overnight. β-Actin (Santa Cruz, Santa Cruz, TX, USA, sc-130656) was used as a loading control. After washing, the membranes were incubated with secondary antibodies (Santa Cruz) and visualized using the ECL (enhance chemiluminescence) Western Blotting Detection System (Tiangen, Beijing, China).

### 4.11. Statistical Analysis

All experiments were performed at least three times in triplicate. Data are presented as mean ± SD of three replications. Statistical significance was assessed with Bonferroni *t*-test in SAS 9.1. Differences were considered statistically significant at *p* < 0.05 (* *p* < 0.05; ** *p* < 0.01).

## 5. Conclusions

We conclude that Sp1 positively regulates *ROCK1* transcription by directly binding to the *ROCK1* core promoter region and may affect the process of myogenesis.

## Supplementary Materials

Supplementary materials can be found at http://www.mdpi.com/1422-0067/17/1/112/s1.
