# Supplementary material for: Transcription Factor Sp1 Promotes the Expression of Porcine ROCK1 Gene
Source: Int J Mol Sci. 2016 Jan 15;17(1):112. doi: 10.3390/ijms17010112 (PMC4730353; doi:10.3390/ijms17010112)
Supplement: Supplementary file 1 [file ijms-17-00112-s001.pdf]

Supplementary Materials: Transcription Factor Sp1 Promotes the Expression of Porcine *ROCK1* Gene

Ruirui Zhang, Xiaoting Feng, Mengsi Zhan, Cong Huang, Kun Chen, Xiaoyin Tang, Tingting Kang, Yuanzhu Xiong and Minggang Lei

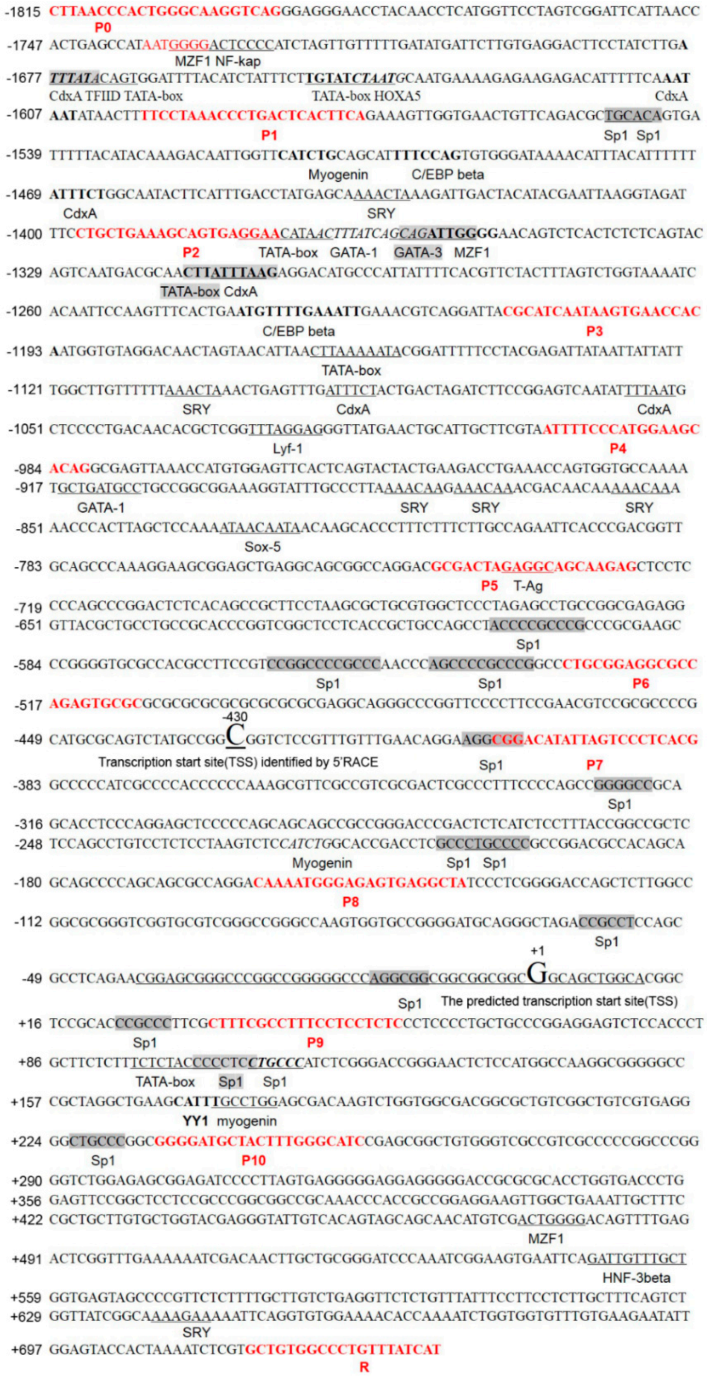

Figure S1. Bioinformatic and experimental analysis of 5′-flanking sequence of porcine *ROCK1* gene. Transcription regulatory motifs obtained from bioinformatic analysis were highlighted with different marks. The primer positions for 5′-deletions were labeled in red color. The transcription start site (TSS) identified by 5′-RCAE and the predicted one were shown in larger font. In addition, the predicted one was set as +1. The Sp1 binding sites in *ROCK1*-P5 were shaded.

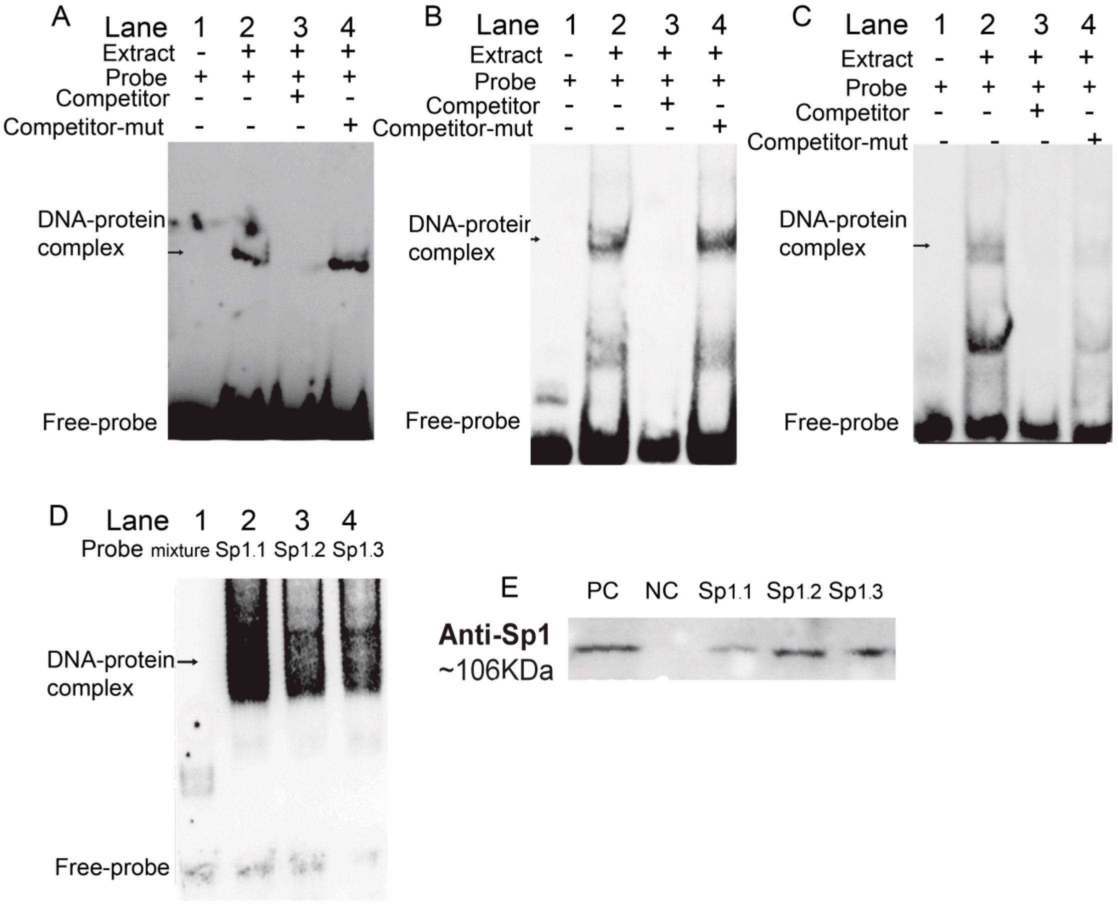

Figure S2. *In vitro* binding of Sp1 with the *ROCK1* promoter was analyzed in LM of pig. (**A**–**C**) The first, second and third biotin-labeled probes were incubated with LM NE under the same condition of PK cells; (**D**) The probes of the three Sp1 binding sites were incubated separately with LM NE; (**E**) Proteins of porcine LM from DNA-pull down materials were examined by Western blot analysis. The three potential Sp1 binding sites were named as Sp1.1-1.3 in (**D**,**E**). The competitor/ competitor-mutant probes were 50-fold excess and arrows indicated the specific DNA—protein complex bands.

Table S1. Primers for 5′RACE, qRT-PCR and ChIP amplification.

Primer Name	Sequence (5′-3′)
UP	CTAATACGACTCACTATAGGGCAAGCA GTGGTATCAACGCAGAGT
NUP	AAGCAGTGGTATCAACGCAGAGT
5′RACE-F	ACAGCGCCGTCGCCACCAGACTT
5′RACE-R	GGACAGGCTGGAGAGCGGCCGGTAA
mus-β-Actin-qRT-PCR-F	GCCTCACTGTCCACCTTCCA
mus-β-Actin-qRT-PCR-R	AGCCATGCCAATGTTGTCTCTT
mus-Sp1-qRT-PCR-F	TCTTCAGGCCCTTCAAGCAG
mus-Sp1-qRT-PCR-R	CTGCCAACTGACCTGTCCAT
mus-ROCK1-qRT-PCR-F	GGCTATTATGGACGAGA
mus-ROCK1-qRT-PCR-R	GAAGGCACAAATGAGAT
mus-MYOD-qRT-PCR-F	GACTTCTATGATGACCCGTGTTTC
mus-MYOD-qRT-PCR-R	TCAGCGTTGGTGGTCTTGC

Table S1. *Cont.*

Primer Name	Sequence (5′-3′)
mus-Myog-qRT-PCR-F	CTACAGGCCTTGCTCAGCTC
mus-Myog-qRT-PCR-R	ACGATGGACGTAAGGGAGTG
mus-MyHC-qRT-PCR-F	CAAGTCATCGGTGTTTGTGG
mus-MyHC-qRT-PCR-R	TGTCGTACTTGGGCGGGTTC
sus-Sp1-qRT-PCR-F	ACCATGAGCGACCAAGATCA
sus-Sp1-qRT-PCR-R	CTGTGTGGCTGTGAGGTCAA
sus-ROCK1-qRT-PCR-F	CCACGTTAAGTGCCACAGAG
sus-ROCK1-qRT-PCR-R	TGTTCATCCTGAGAACATGC
ChIP-Sp1-F	CACCGCTGCCAGCCTAC
ChIP-Sp1-R	ACTCTGGCGCCTCCGCA

Table S2. Primers for 5′-deletation of porcine *ROCK1* promoter and Sp1 CDS amplification.

Primer Name	Sequence (5′-3′)
ROCK1-P0	CTTAACCCACTGGGCAAGGTCAG
ROCK1-R	ATGATAAACAGGGCCACAGC
ROCK1-P0-KpnI	GGGGTACCCTTAACCCACTGGGCAAGGTCAG
ROCK1-P1-KpnI	GGGGTACCTTCCTAAACCCTGACTCACTTCA
ROCK1-P2-KpnI	GGGGTACCCTGCTGAAAGCAGTGAGGAA
ROCK1-P3-KpnI	GGGGTACCCGCATCAATAAGTGAACCACA
ROCK1-P4-KpnI	GGGGTACCATTTTCCCATGGAAGCACAG
ROCK1-P5-KpnI	GGGGTACCGCGACTAGAGGCAGCAAGAG
ROCK1-P6-KpnI	GGGGTACCCTGCGGAGGCGCCAGAGT
ROCK1-P7-KpnI	GGGGTACCCGGACATATTAGTCCCTCACG
ROCK1-P8-KpnI	GGGGTACCCAAAATGGGAGAGTGAGGCTA
ROCK1-P9-KpnI	GGGGTACCCTTTCGCCTTTCCTCCTCTC
ROCK1-P10-KpnI	GGGGTACCGGGATGCTACTTTGGGCATC
ROCK1-R-Hind III	CCCAAGCTTATGATAAACAGGGCCACAGC
Sp1-F	CCCAAGCTTATGAGCGACCAAGATCACTCC
Sp1-R	GGGGTACCTCAGAAGCCATTGCCACTGAT

Table S3. Oligo sequences used for EMSA and site-directed mutation.

Oligo Name	Sequence (5′-3′)
Sp1-1-F-bio	CTGCCAGCCTACCCCGCCCGCCCGCGAAGC-bio
Sp1-1-R-bio	GCTTCGCGGGCGGGCGGGGTAGGCTGGCAG-bio
Sp1-1-F	CTGCCAGCCTACCCCGCCCGCCCGCGAAGC
Sp1-1-R	GCTTCGCGGGCGGGCGGGGTAGGCTGGCAG
Sp1-Mut-1-F	CTGCCAGCCTACCCCTCCCGCCCGCGAAGC
Sp1-Mut-1-R	GCTTCGCGGGCGGGAGGGGTAGGCTGGCAG
Sp1-2-F-bio	CGCCTTCCGTCCGGCCCCGCCCAACCCAGC-bio
Sp1-2-R-bio	GCTGGGTTGGGCGGGGCCGGACGGAAGGCG-bio
Sp1-2-F	CGCCTTCCGTCCGGCCCCGCCCAACCCAGC
Sp1-2-R	GCTGGGTTGGGCGGGGCCGGACGGAAGGCG
Sp1-Mut-2-F	CGCCTTCCGTCCGGTTTGCCCAACCCAGC

Table S3. *Cont.*

**Oligo Name**	**Sequence (5′-3′)**
Sp1-Mut-2-R	GCTGGGTTGGGCGAAACCGGACGGAAGGCG
Sp1-3-F-bio	CGCCCAACCCAGCCCCGCCCGGCCCTGCGG-bio
Sp1-3-R-bio	CCGCAGGGCCGGGCGGGGCTGGGTTGGGCG-bio
Sp1-3-F	CGCCCAACCCAGCCCCGCCCGGCCCTGCGG
Sp1-3-R	CCGCAGGGCCGGGCGGGGCTGGGTTGGGCG
Sp1-Mut-3-F	CGCCCAACCCAGCTTTGCCCGGCCCTGCGG
Sp1-Mut-3-R	CCGCAGGGCCGGGCAAAGCTGGGTTGGGCG

The series of Mut Oligo sequences were also used for site-directed mutation of the Sp1 binding sites in another type of purification.
